# Secondary analysis of hospital patient experience scores across England’s National Health Service – How much has improved since 2005?

**DOI:** 10.1371/journal.pone.0187012

**Published:** 2017-10-26

**Authors:** Kate Honeyford, Felix Greaves, Paul Aylin, Alex Bottle

**Affiliations:** 1 Dr Foster Unit at Imperial College, London, England; 2 Department of Primary Care and Public Health, Imperial College, London, England; Maastricht Universitair Medisch Centrum+, NETHERLANDS

## Abstract

**Objective:**

To examine trends in patient experience and consistency between hospital trusts and settings.

**Methods:**

Observational study of publicly available patient experience surveys of three hospital settings (inpatients (IP), accident and emergency (A&E) and outpatients (OP)) of 130 acute NHS hospital trusts in England between 2004/05 and 2014/15.

**Results:**

Overall patient experience has been good, showing modest improvements over time across the three hospital settings. Individual questions with the biggest improvement across all three settings are cleanliness (IP: +7.1, A&E: +6.5, OP: +4.7) and information about danger signals (IP: +3.8, A&E: +3.9, OP: +4.0). Trust performance has been consistent over time: 71.5% of trusts ranked in the same cluster for more than five years. There is some consistency across settings, especially between outpatients and inpatients. The lowest-scoring questions, regarding information at discharge, are the same in all years and all settings.

**Conclusions:**

The greatest improvement across all three settings has been for cleanliness, which has seen national policies and targets. Information about danger signals and medication side-effects showed least consistency across settings and scores have remained low over time, despite information about danger signals showing a big increase in score. Patient experience of aspects of access and waiting have declined, as has experience of discharge delay, likely reflecting known increases in pressure on England’s NHS.

## Introduction

Patient experience is increasingly seen as an important aspect of healthcare, both as an ‘intrinsically important dimension of care quality [1], and stimulus for improvement [2], and the last 20 years have seen a proliferation of national patient experience surveys in many countries[3]. Patient experience scores have shown associations with several outcomes including adherence to medication [4], good clinical process measures [5] and fewer inpatient care complications [6], although some dispute the link’s causality [7].

The National Health Service (NHS) National Patient Survey Programme, administered by Picker Institute Europe, covers patients’ experiences of a range of health provision. The Care Quality Commission (CQC), the national regulator, reports results for each trust [8]. In a Diagnostic Tool [9] published by the Department of Health (DoH) the questions in the three main hospital surveys are partitioned into five key domains (Box 1). The DoH suggest the tool shows how scores vary across NHS healthcare providers for both NHS managers and the general public.

**Box 1 Five domains of patient experience**

- Access and waiting (AW) e.g. *How long did you wait before you first spoke to a nurse or doctor?*
- Safe; high quality; co-ordinated care (SHQCC) e.g. *Did a member of staff tell you about any danger signals regarding your illness or treatment to watch for after you went home?*
- Better Information, More Choice (BIMC) e.g. *Were you involved as much as you wanted to be in decisions about your care and treatment?*
- Building closer relationships (BR) e.g. *Did doctors or nurses talk in front of you as if you weren't there? Did doctors or nurses talk in front of you as if you weren't there?*
- Clean; friendly; comfortable place to be (CCFP) e.g. *Overall; did you feel you were treated with respect and dignity while you were in the hospital?*

The domains have similar questions and scoring methodologies across different settings (inpatient (IP), Accident and Emergency (A&E) and outpatient (OP)) and are combined into an Overall Patient Experience Score (OPES). Each domain score is the mean of the scores for question within the domain, the OPES is the mean of the five domain scores. The questions included in the domains are unchanged since the surveys’ inception, making them ideal for looking at trends over time and between settings.

There are now ten continuous years of inpatient patient experience data as well as additional surveys of A&E and outpatient departments, with approximately one million patients responding. Given the importance of patient experience within the NHS, the annual publication of results and the repetition of the questions being asked we would hope scores to improve over time and performance between settings to become more consistent.

A recent report on inpatients [10] highlights the overall positive experience, with ongoing improvements, especially in areas of policy intervention. Negative trends were seen in areas where ‘there are well-recognised pressures in the system’. Trust-level inpatient analysis suggested that the majority of trusts have not improved consistently (a trust can comprise several hospitals) [11]. Analysis of the 2008 and 2009 inpatient, outpatient and A&E surveys using cluster analysis found that 21% of trusts had above-average performance across all surveys and all domains of care, but only 4% of trusts were above- or below-average performance across all three settings (A&E, OP and IP) suggesting that trusts do not perform consistently across settings [12].

Our study extends this recent work by analysing trends over time in three key hospital settings (inpatient, outpatient and A&E), comparing a trust’s performance with other trusts and comparing hospital settings. We aim to determine if:

1. the patient experience in each setting has changed over time,
2. trusts have performed consistently over time and,
3. there is consistency between hospital settings.

## Methods

This study utilises publicly available results of NHS patient surveys completed between 2004/05 and 2014/15. Details of sample sizes and response rates are available from the NHS Surveys website; summary tables (S1 Table and S2 Table) are include in supporting information. Inpatient surveys are annual. There have been five A&E and four outpatient surveys since 2003. In 2003 and 2004/05 these were in the same year, but since then they have been in different years. The most recent surveys included in the analysis for A&E and OP patients were 2014/15 and 2011/12 respectively.

Prior to publication, scores are standardised based on age, gender and, for inpatients, route of admission [13].

We included 130 acute hospital trusts with inpatient survey results for the ten-year period 2005/06 to 2014/15. The majority of trusts which had data for some years but not others were trusts which were merged or were newly formed during the period of study. Specialist trusts which generally have a single speciality were excluded; they were the highest scoring trusts for IP surveys in all years.

Initially descriptive analysis of data from NHS England’s Patient Experience Tool [9] determined the patterns in scores over time for overall patient experience, domains and individual questions, for inpatients, outpatients and A&E. Scores in 2005/06 and 2014/15 were also compared. To determine if performance of the highest- and lowest-scoring trusts was consistent over time, the mean score for each trust and domain in the first three years was calculated and the 25% highest-scoring and 25% lowest-scoring trusts were identified. The mean scores for these groups of trusts were then calculated for each year.

To assess performance consistency, trusts’ performances over time and relative to one another were analysed. Trusts were grouped into four using k-means cluster analysis using standardised patient experience scores. Although Ward’s minimum variance hierarchical clustering [14] suggested different numbers of clusters in different years, ranging from four to nine, four was selected as a pragmatic approach. Consistent performance was defined as being in the same ranked cluster for greater than five years.

To assess consistency between hospital settings within hospitals, A&E scores from 2014/15 were compared with the same year’s inpatient scores. Outpatient scores from 2011/12 were separately compared with inpatient scores for the same year. Cluster analysis determined consistency across settings. Trusts were grouped into four clusters based on A&E or outpatient scores and these were compared with clusters based on their inpatient scores. In addition, trusts were divided into quartiles. This was completed for OPES (the overall score), five domains and seven questions with identical wording across the three surveys.

To identify trusts which had improved over time we compared the mean inpatient score for the first three years of the inpatient survey and compared this to the mean score the final three years of the study period. The 10% of trusts which had made the biggest improvements were identified. Similarly, we identified the 10% of trusts for whom their scores had improved the least. The trusts which had improved the most were compared with the trusts which had improved the least in terms of bed numbers, bed occupancy and staffing levels.

Sensitivity analyses were carried out on the impact of the clustering approach on the consistency over time: Ward’s minimum variance hierarchical clustering, k-means using different seeds and a simple quartile approach. There were minor impacts on the results when five outliers were removed, which would not affect conclusions. The outliers were therefore retained in the main analyses. All analysis was done using SAS v9.4.

## Results

### Trends in patient experience scores over time

**Fig 1:**
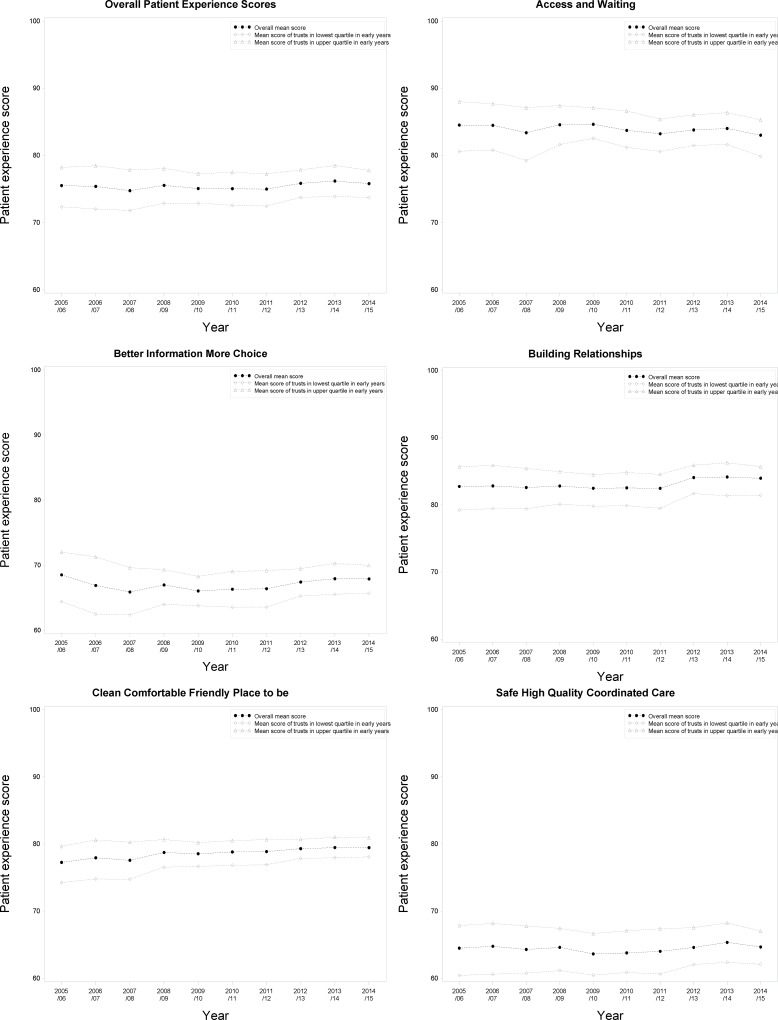
Trends in patient experience scores for inpatients over time, overall (OPES) and by domain. Mean scores for the 25% lowest scoring trusts and the 25% highest scoring trusts in the first three years are also shown.

Fig 1 shows trends in domain and overall inpatient scores. There has been a steady two-point increase in the Clean, Comfortable, Friendly Place to be (CCFP) domain. Other domains have more erratic patterns, especially Safe, High Quality, Co-ordinated Care (SHQCC). Access and Waiting (AW) scores fell overall but with major fluctuations. Both Better Information, More Choice (BIMC) and Building Relationships (BR) saw relatively small improvements between 2011/12 and 2012/13.

Table 1 summarises changes in inpatient, outpatient and A&E scores over time. Outpatient experience improved across all domains between 2004/05 and 2011/12, especially for AW. Overall A&E scores improved between 2004/05 and 2014/15, with the biggest increase for CCFP. There was a decline in AW scores for A&E patients. Changes in outpatient and A&E scores were greater than for inpatients.

**Table 1:** Summary of changes over time in scores by department and domain for all acute non-specialist English hospital trusts.

Domain/Question	Mean score in 2005/06 (variance)	Mean score 2014/15 (variance)	Mean change in score between 2005/06 and 2014/15 (variance)
INPATIENT SCORES			
**Overall Patient Experience Score (OPES)**	76.2 (3.56^2^)	76.6 (3.68^2^)	0.8 (1.79^2^)
**Access and waiting (AW)**	85.2 (3.82^2^)	83.8 (4.15^2^)	-0.4 (2.16^2^)
**Safe; high quality; co-ordinated care (SHQCC)**	65.4 (4.61^2^)	65.6 (4.64^2^)	0.5 (2.65^2^)
**Better Information; more choice (BIMC)**	69.3 (4.42^2^)	68.9 (4.69^2^)	0.8 (2.88^2^)
**Building closer relationships (BR)**	83.3 (3.28^2^)	84.6 (3.21^2^)	1.4 (1.48^2^)
**Clean; friendly; comfortable place to be (CCFP)**	78.0 (3.43^2^)	80.1 (3.1^2^)	1.8 (1.89^2^)
**ACCIDENT AND EMERGENCY**	**Mean score in 2004/05 (variance)**	**Mean score 2014/15 (variance)**	**Mean change in score between 2004/05 and 2014/15 (variance)**
**Overall Patient Experience Score (OPES)**	76.0 (3.08^2^)	77.2 (2.92^2^)	1.2 (3.29^2^)
**Access and waiting (AW)**	69.6 (4.76^2^)	67.8 (4.39^2^)	-1.8 (4.94^2^)
**Safe; high quality; co-ordinated care (SHQCC)**	75.0 (3.51^2^)	76.1 (3.35^2^)	1.1 (3.74^2^)
**Better Information; more choice (BIMC)**	73.6 (3.43^2^)	76.0 (3.58^2^)	2.4 (4.23^2^)
**Building closer relationships (BR)**	80.5 (2.81^2^)	81.9 (2.84^2^)	1.4 (3.32^2^)
**Clean; friendly; comfortable place to be (CCFP)**	81.2 (3.29^2^)	84.3 (2.94^2^)	3.1 (3.70^2^)
**OUTPATIENT DEPARTMENT**	**Mean score in 2004/05 (variance)**	**Mean score 2011/12 (variance)**	**Mean change in score between 2004/05 and 2011/12 (variance)**
**Overall Patient Experience Score (OPES)**	75.6 (2.25^2^)	79.2 (1.91^2^)	2.5 (1.38^2^)
**Access and waiting (AW)**	69.1 (2.99^2^)	75.1 (2.75^2^)	6.0 (2.34^2^)
**Safe; high quality; co-ordinated care (SHQCC)**	82.2 (2.33^2^)	83.5 (2.03^2^)	1.3 (1.83^2^)
**Better Information; more choice (BIMC)**	77.0 (3.08^2^)	78.5 (2.58^2^)	1.5 (2.56^2^)
**Building closer relationships (BR)**	86.3 (1.88^2^)	87.5 (1.87^2^)	1.2 (1.49^2^)
**Clean; friendly; comfortable place to be (CCFP)**	68.4 (3.21^2^)	71.0 (2.59^2^)	2.6 (2.61^2^)

Individual questions with the biggest improvement are the same across all three settings: cleanliness (IP: +7.1, A&E: +6.5, OP: +4.7) and information about danger signals (IP: +3.8, A&E: +3.9, OP: +4.0). Outpatients saw an improvement in both dimensions of AW, particularly for total waiting time (+8.4). A&E patients reported the biggest improvement in experience of information about medication (both purpose (+3.3) and side-effects (6.7)), as well as pain control (+4.4) and time to discuss health problems (+3.4). A&E patients’ experience of waiting to speak to a nurse or doctor fell the most (-5.6), followed by inpatients’ experience of waiting for a bed (-4.4) and discharge delay (-3.8).

The majority of trusts (67%) improved their overall inpatient score between 2005/06 and 2014/15, although the mean change is less than 1. The BR and CCFP domains had the highest percentages of trusts that improved. Over 50% of trusts have a lower AW score in 2014/15 than 2005/06. The majority of trusts improved across all domains for both outpatient and A&E departments, except the AW domain for A&E. The variance in inpatient scores did not fall over time. There is evidence that the variance in outpatient scores fell between 2004/05 and 2011/12. The lowest- and highest-scoring questions have remained consistent over all the years of the survey in all three settings.

### Consistency in trust performance over time–inpatient experience

Consistency in inpatient scores over the ten years was high (Table 2). 71.5% of trusts were in the same ranked cluster for more than five of the ten years for overall scores. There was also high consistency for individual domains.

**Table 2:** Consistency of trust performance ranking in inpatient experience scores between 2005/06 and 2014/15 for acute non-specialist trusts in England.

	Number of trusts (percentage) in same ranked cluster for greater than five years.	Number of trusts (*percentage*) in the highest performing cluster for greater than five years.	Number of trusts (*percentage*) in the second highest performing cluster for greater than five years.	Number of trusts (*percentage*) in the third highest performing cluster for greater than five years.	Number of trusts (*percentage*) in the lowest performing cluster for greater than five years.
Inpatient domains					
**Overall Patient Experience Score (OPES)**	93 *(71*.*5%)*	19 *(14*.*6%)*	35 *(26*.*9%)*	32 *(24*.*6%)*	7 *(5*.*4%)*
**Access and waiting (AW)**	83 *(63*.*8%)*	19 *(14*.*6%)*	38 *(29*.*2%)*	27 *(20*.*8%)*	4 *(3*.*1%)*
**Safe; high quality; co-ordinated care (SHQCC)**	92 (*70*.*8%*)	11 (*8*.*5%*)	38 (*29*.*2%*)	38 (*29*.*2%*)	5 (*3*.*8%*)
**Better Information; more choice (BIMC)**	72 *(55*.*4%)*	16 *(12*.*3%)*	25 *(19*.*2%)*	26 *(20*.*0%)*	5 *(3*.*8%)*
**Building closer relationships (BR)**	104 *(80*.*0%)*	28 *(21*.*5%)*	48 *(36*.*9%)*	24 *(18*.*5%)*	6 *(4*.*6%)*
**Clean; friendly; comfortable place to be (CCFP)**	94 (*72*.*3%*)	17 (*13*.*1%*)	40 (*30*.*8%*)	29 (*22*.*3%*)	8 (*6*.*2%*)

The gap between the lowest- and highest-performing trusts in the initial period narrowed during the first three years, but there was little evidence of the lowest-performing trusts ‘catching up’ after this, except for the CCFP domain (Fig 1).

### Consistency in trust performance across settings

Questions regarding waiting and information about medication side-effects and danger signals have been consistently low-scoring in all three settings since the survey inception. High-scoring questions also show consistency over time and across settings and include being treated with respect and dignity and being given sufficient privacy.

Using cluster and quartile analysis to determine performance consistency across settings, approximately 50% of trusts were in the same cluster for A&E or OP and inpatient surveys overall (Table 3). In general, consistency was higher between OP and inpatients than between A&E and inpatients. Consistency was lower for individual domains and individual questions. Cleanliness scores had the highest consistency across settings. Lowest consistency was seen for the lowest-scoring question, receiving information about medication side-effects. Changes over time varied between domains and questions; however, high scores on many questions reduce what variation is possible.

**Table 3:** Consistency between inpatient and outpatient or A&E patient experience scores for domains and questions that are identically worded in all three surveys.

	Number of trusts (percentage) in the same ranked group–A&E and inpatient scores (2014/15).			Number of trusts (percentage) in the same ranked group–outpatient and inpatient scores (2011/12).		
	Quartiles	Cluster analysis		Quartiles	Cluster analysis	
	2014/15	2014/15	2008/09	2011/12	2011/12	2009/10
**Overall Patient Experience Score (OPES)**	63 (48.5%)	66 (50.8%)	64 (49.2%)	67 (51.5%)	72 (55.4%)	77 (59.2%)
**Access and waiting (AW)**	47 (36.2%)	51 (39.2%)	53 (40.8%)	49 (37.7%)	34 (26.2%)	60 (46.2%)
**Safe; high quality; co-ordinated care (SHQCC)**	56 (43.1%)	56 (43.1%)	45 (34.6%)	55 (42.3%)	54 (41.5%)	66 (50.8%)
**Better Information; more choice (BIMC)** *Missing data for aeBIMC 2014/15*^*1*^	46 (38.7%)	51 (42.9%)	50 (38.5%)	45 (34.6%)	53 (40.8%)	78 (60.0%)
**Building closer relationships (BR)**	46 (35.4%)	57 (43.8%)	55 (42.3%)	46 (35.4%)	54 (41.5%)	83 (63.8%)
**Clean; friendly; comfortable place to be (CCFP)**	49 (37.7%)	49 (37.7%)	59 (45.4%)	49 (37.7%)	49 (37.7%)	67 (51.5%)
**SHQCC1 Sometimes a member of staff will say one thing and another will say something quite different. Did this happen to you? (aeSHQCC1; opSHQCC4)**	39 (30.0%)	46 (35.4%)	55 (42.3%)	*39 (30*.*0%)*	52 (40.0%)	58 (44.6%)
**SHQCC3 Did a member of staff tell you about any danger signals you should watch for after you went home? (aeSHQCC2; opSHQCC5)**	42 (32.3%)	44 (33.8%)	46 (35.4%)	42 (32.3%)	55 (42.3%)	58 (44.6%)
**BIMC1**^**2**^ **Were you involved as much as you wanted to be in decisions made about your care and treatment? (aeBIMC1; opBIMC2)**	51 (39.2%)	57 (43.8%)	52 (40.0%)	51 (39.2%)	72 (55.4%)	44 (33.8%)
**BIMC2**^**3**^ **Did a member of staff explain the purpose of the medications you were to take home in a way you could understand? (aeBIMC2; opBIMC4)**	43 (33.6%)	49 (32.0%)	63 (48.5%)	43 (33.1%)	31 (23.8%)	55 (42.3%)
**BIMC3**^**4**^ **Did a member of staff tell you about medication side effects to watch for when you went home? (aeBIMC3; opBIMC5)**	35 (29.2%)	45 (37.5%)	46 (35.4%)	35 (26.9%)	44 (33.8%)	54 (41.5%)
**CCFP3 In your opinion how clean was the hospital room or ward that you were in/Emergency Department/ Outpatients Department? (aeCCFP3; opCCFP2)**	59 (45.4%)	60 (46.2%)	60 (46.2%)	59 (45.4%)	68 (52.3%)	78 (60.0%)
**CCFP6 Overall did you feel you were treated with respect and dignity while you were in hospital/Emergency Department/Outpatients Department? (aeCCFP4; opCCFP3)**	45 (34.6%)	59 (45.4%)	45 (34.6%)	45 (34.6%)	53 (40.8%)	50 (38.5%)

^1^aeBIMC: n = 119; ^2^aeBIMC1: n = 130; ^3^aeBIMC2: n = 128; ^4^aeBIMC3: n = 120

### Improving trusts

The 10% of trusts which had made the biggest improvements were both low and high performing trust in the 2005/06. There was no evidence of patterns in the geographical location or types of trusts. Two trusts were in the lowest ranked cluster, three were in the highest ranked cluster and the remaining eight trusts were in the middle ranked clusters. Two trusts were also improvers in terms of A&E PE scores, three in terms of OP scores and two in both A&E and OP scores. Table 4 summarises the characteristics of the most and least improving trusts. There is limited evidence that the most improving trusts have a higher number of beds, and a higher number of doctors and consultants per 10 beds; p>0.5 for all comparisons.

**Table 4:** Mean characteristics of most improving trusts and least improving trusts.

Characteristics of trusts (2010/11)	Most improving trusts (median (IQR))	Least improving trusts (median (IQR))
**Mean number of nurses per 10 beds**	1.63 (1.53 to 1.76)	1.70 (1.62 to 1.79)
**Mean number of doctors per 10 beds**	0.71 (0.58 to 0.77)	0.64 (0.58 to 0.68)
**Mean number of consultants per 10 beds**	0.25 (0.20 to 0.27)	0.22 (0.21 to 0.28)
**Mean number of beds**	644 (572 to 750)	573 (446 to 957)
**Mean bed occupancy (%)**	89% (84% to 91%)	89% (84% to 90%)

## Discussion

### Main findings

During 11 years of national NHS hospital surveys, overall patient experience (PE) has been consistently high in most areas, with minor increases in the majority of scores. PE of access and waiting (AW) for both A&E and inpatients has declined, but outpatient scores have risen. *Scores for the ‘Clean; friendly; comfortable place to be’ (CCFP) domain have improved across all three settings (IP*:*+1*.*8*, *A&E*: *+3*.*1 and OP*:*+2*.*6)*, *mainly attributable to increases in PE of cleanliness*. Experience of waiting and information at discharge were low-scoring in all years in all three settings; a similar pattern was seen for high-scoring questions. PE of cleanliness has shown a marked improvement in all three settings, as has information about danger signals. Outpatient scores improved for waiting for an appointment, whilst waiting for a bed and discharge delay has deteriorated for inpatients, as has waiting to speak to a nurse or doctor for A&E patients. Trusts’ performance in comparison with each other has also been consistent over time and between settings. Inspection of the trusts which improved the most and the least did not identify any patterns in area or types of trust. There is some evidence that the most improving trusts had a higher supply of doctors and consultants but not nursing staff.

### Strengths and limitations

This study is the most comprehensive summary of detailed, national NHS patient survey data across three settings since its inception. We focused on domains developed by the Department of Health which have included the same questions since the initial surveys. Although other domains have been suggested and there may be challenges with combining questions into domains, the consistency of the questions over time and between trusts means this is a pragmatic approach.

Comparing PE across the three hospital settings has inherent difficulties as patients’ expectations will vary by department, possibly influencing their responses on PE surveys [14, 15].

Cluster analysis assigns trusts to groups based on actual variation in performance, in contrast to dividing trusts into quartiles which are inherently unstable [16]. The consistency measure depends on the method used and the number of clusters selected, but similar trends were seen with a quartile approach.

Excluding trusts which did not have complete data for the 10 years meant excluding trusts which merged or newly formed during the ten years of study. Mean scores of trusts without complete data were typically lower than the mean, but it is not clear why this is the case. A separate study of these trusts would provide information the impact of merging on patient experience of mergers.

Changes in the scores are modest. Whether this is because PE has actually changed little or because the survey instruments are insensitive to real improvements in care cannot be distinguished from the survey results alone. In addition, trusts which are already performing well may find it hard to improve as the majority of patients are also scoring the maximum (ceiling effect). Lastly, for many questions there are only two options if the patient is reasonably happy, with an emphasis on always or often, which may pose another challenge for trusts wishing to improve.

Identifying features of trusts which show the biggest improvements has not revealed clear patterns. Limited trust data was available for this analysis. For example, a review of trust annual reports and websites to identify trust level initiative may reveal common approaches in the most improving trusts. This was beyond the scope of this study.

### Implications

Previous analysis of the 2009 inpatient and outpatient and 2008 A&E surveys suggested that 21% trusts consistently performed above or below average with lower levels of consistency between hospital settings [12]. We found higher consistency between settings, which might be due to different domains of care or the number of clusters used. Our research reinforces the finding that trusts perform consistently relative to each other, not just across domains but also over time and between settings.

We found that the big improvements in inpatient cleanliness scores [10, 17] were also seen in the outpatient and A&E surveys. These improvements coincided with national targets and campaigns such as the NHS ‘cleanyourhands’ campaign [18]. It has been suggested that the biggest improvements are in areas of policy intervention such as cleanliness [10, 19]. Information about danger signals has also shown big improvements across all three settings with no evidence of an associated national intervention. These improvements may be due to action following the low scores.

Both the inpatient and outpatient scores improved for waiting time for an appointment, which mirrors a reduction in waiting time between referral and treatment seen in other data [20]. Similarly, A&E patients report a worsening of experience in waiting to speak to a doctor or nurse, reflected in an increase in time to initial assessment since 2011 [20]. The agreement between PE waiting measures and other waiting time data provides good evidence that PE surveys are useful barometers of waiting time performance.

The consistency in low-scoring questions covering “medication side-effects” and “danger signals to look out for” in all surveys suggest that these surveys can inform trusts about areas requiring trust-wide action. This partly counters concerns that trust-wide surveys do not reflect what happens in individual departments.

Possible barriers to using data more effectively have been proposed [10, 21] and include a lack of time, delays in dissemination, the introduction of the Friends and Family Test, scepticism among clinicians and limited understanding of statistical methods. There has been no systematic review of the way in which trusts have used the results, although this need has been highlighted [19], as has the need for systematic guidance on how to use data [22]. One potential use is the evaluation of initiatives such as ‘Hello My Name is…’ [23].

## Conclusion

Despite the pressures on the NHS over the last ten years, there is strong evidence that patients’ experiences of hospitals are positive and that they are generally satisfied with the care they receive. Key areas of improvement include the policy-driven improvement in cleanliness scores in all three settings. PE of aspects of access and waiting have declined, as has experience of discharge delay, likely reflecting known increases in pressure on England’s NHS. Information about danger signals and medication side-effects showed least consistency in all settings and scores have remained low over time. The use of patient surveys to improve patient experience and subsequent quality improvement in the NHS need to be developed.

## Supporting information

S1 Table Number of respondents to NHS hospital surveys and response rates (%). (DOCX)

S2 Table Proportion of respondents by sex and age in most recent surveys. (DOCX)
