# Supplementary material for: Secondary analysis of hospital patient experience scores across England’s National Health Service – How much has improved since 2005?
Source: PLoS One. 2017 Oct 26;12(10):e0187012. doi: 10.1371/journal.pone.0187012 (PMC5658118; doi:10.1371/journal.pone.0187012)
Supplement: S1 Table — (DOCX) [file pone.0187012.s001.docx]

**Supplementary Information**

**Survey response rates and demographics**

|  | **Inpatient** | **A&E** | **Outpatient** |
| --- | --- | --- | --- |
| **2002** | 95, 280 (64%)*^1^ |  |  |
| **2003** |  | 59,155 (46%)*^2^ | 90 552 (63%)*^2^ |
| **2004/05** |  | 55,339 (44%) | 84,280 (59%) |
| **2005/06** | 80,793 (59%) |  |  |
| **2006/07** | 80,694 (59%) |  |  |
| **2007/08** | 75,949 (56%) |  |  |
| **2008/09** | 72,584 (54%) | 49,646 (40%) |  |
| **2009/10** | 69,348 (52%) |  | 71,827 (53%) |
| **2010/11** | 66,348 (50%) |  |  |
| **2011/12** | 70,863 (53%) |  | 72, 773 (53%) |
| **2012/13** | 64,505 (51%) | 45,594 (38%) |  |
| **2013/14** | 62,443 (49%) |  |  |
| **2014/15** | 59,083 (47%) | 40,000 (34%) |  |

Table S1 Number of respondents to NHS hospital surveys and response rates (%).

*^1^Department of Health carried out the survey

*^2^Commission for Health Improvement (CHI) carried out the survey
