# Supplementary material for: Secondary analysis of hospital patient experience scores across England’s National Health Service – How much has improved since 2005?
Source: PLoS One. 2017 Oct 26;12(10):e0187012. doi: 10.1371/journal.pone.0187012 (PMC5658118; doi:10.1371/journal.pone.0187012)
Supplement: S2 Table — (DOCX) [file pone.0187012.s002.docx]

**Supplementary Information**

| **Survey** | **Proportion of respondents who are male** | **Proportion of respondents aged:** | | | |
| --- | --- | --- | --- | --- | --- |
|  |  | **16-35** | **36-50** | **51-65** | **66 or older** |
| **Inpatients 2013/14^1^** | 46% | 7% | 12% | 24% | 57% |
| **Inpatients 2014/15^1^** | 47% | 6% | 11% | 23% | 59% |
| **Outpatient 2009/10^2^** | 43% | 9% | 16% | 29% | 46% |
| **Outpatient 2011/12^2^** | 43% | 8% | 15% | 29% | 48% |
| **A&E 2012/13^3^** | 45% | 17% | 19% | 24% | 40% |
| **A&E 2014/15^3^** | 45% | 15% | 17% | 24% | 45% |

Table S2 Proportion of respondents by sex and age in most recent surveys.

^1^CQC NHS. National NHS patient survey programme. National results from the 2014 Inpatient Survey. <http://www.nhssurveys.org/Filestore/National_results_from_the_2014_Inpatient_survey.pdf>

^2^National NHS patient survey programme - Outpatient department survey 2011 – Full national results with historical comparisons [www.cqc.org.uk/content/outpatient-survey-2011](http://www.cqc.org.uk/content/outpatient-survey-2011)

^3^CQC. National NHS patient survey programme Accident and Emergency Survey 2014 [www.cqc.org.uk/sites/default/files/20141201_accident_and_emergency_survey_2014_national_tables.pdf](http://www.cqc.org.uk/sites/default/files/20141201_accident_and_emergency_survey_2014_national_tables.pdf)
